# Zinc Deficiency Induces Autophagy in HT-22 Mouse Hippocampal Neuronal Cell Line

**DOI:** 10.3390/ijms23158811

**Published:** 2022-08-08

**Authors:** Si-Yeon Kim, Jung-Ho Lee, Soon-Ae Kim

**Affiliations:** Department of Pharmacology, School of Medicine, Eulji University, Daejeon 34824, Korea

**Keywords:** zinc, zinc deficiency, autophagy, AMPK, mTOR, SIRT1

## Abstract

Zinc is a trace metal vital for various functions in nerve cells, although the effect of zinc deficiency on neuronal autophagy remains unclear. This study aimed to elucidate whether zinc deficiency induced by treatment with N, N, N′, N′-tetrakis (2-pyridylmethyl) ethylenediamine (TPEN), a zinc chelator, affects and alters autophagy activity. In cell viability assays, TPEN showed cytotoxicity in HT-22 cells. TPEN treatment also increased LC3-II levels and the ratio of LC3-II to LC3-I. Western blot analysis showed that phospho-AMP-activated protein kinase levels and the ratio of phospho-AMP-activated protein kinase to total AMP-activated protein kinase increased. Protein levels of the mammalian target of rapamycin and sirtuin 1 decreased following TPEN treatment. When TPEN-treated HT-22 cells were cotreated with autophagy inhibitors, 3-methyladenine (1 mM), or bafilomycin A1 (3 nM), the TPEN-induced decrease in cell viability was exacerbated. Cotreatment with chloroquine (10 μM) partially restored cell viability. The study showed that zinc deficiency induces autophagy and may be cytoprotective in neurons. We expect our results to add a new perspective to our understanding of the neuronal pathology related to zinc deficiency.

## 1. Introduction

Zinc is the second most abundant trace metal after iron and plays an essential role in various cellular functions, including transcription, cell signaling pathways, and enzyme activity [1]. The brain has an exceptionally high zinc concentration. Most of the zinc in the brain is protein-bound, and zinc is highly localized in the mossy fiber terminals of the dentate gyrus [2]. Approximately 20% of the global population suffers from dietary zinc deficiency [3]. Several studies have demonstrated that zinc deficiency is associated with cognitive decline [4]. Studies using animal models have also reported that zinc deficiency impairs learning and memory [5,6,7].

Autophagy is a conserved process and is a well-known mechanism by which cells adapt to stress by degrading intracellular components. Given that autophagy plays a vital role in cellular homeostasis, it seems reasonable that autophagy plays an important role when zinc levels are below physiological levels. Approximately 40 autophagy-related genes (ATG) are involved in autophagy and complex regulatory mechanisms, including AMP-activated protein kinase (AMPK) and mammalian target of rapamycin (mTOR), alter their activity. AMPK significantly affects neuronal homeostasis. AMPK senses low intracellular ATP levels and adapts to cellular metabolism and autophagy [8]. AMPK and mTOR regulate autophagy initiation [9]. mTOR, a serine/threonine kinase, is a master regulator of cellular metabolism and the most widely studied regulator of autophagy. Among the two mTOR-including signaling complexes, mTORC1 regulates cell growth, proliferation, and autophagy induction [10]. A recent study has shown that zinc depletion induces autophagy by inhibiting TORC1 in yeast [11].

Recently, many researchers have conducted studies on the effect of zinc on the autophagy process; however, the results have been inconsistent. Some studies have reported that high zinc levels and its ionophores stimulate autophagy [12,13]. However, reports on the effects of zinc deficiency on autophagy are complex. Some studies using the zinc chelator N, N, N′, N′-tetrakis (2-pyridylmethyl) ethylenediamine (TPEN) after various autophagy-inducing conditions, such as ischemia, have shown that zinc deficiency restores previously induced autophagy [14,15]. However, studies on zinc deficiency models without prior stress exposure to autophagy-related conditions are still lacking. We identified only two studies in yeast, which suggested that zinc deficiency induces autophagy [11,16]. The effect of zinc deficiency on autophagy has not been well studied. Zinc deficiency may cause autophagy in mammalian cells, depending on the cell type [17]. Since the hippocampus contains a large amount of zinc, it is crucial to understand how zinc deficiency affects hippocampal neurons. However, no studies have investigated how zinc deficiency affects hippocampal neuronal autophagy and the important autophagy regulators, mTOR, and AMPK signaling. Therefore, the present study aimed to investigate the effect of zinc deficiency on autophagy in hippocampal neurons and its action mechanism.

## 2. Results

### 2.1. Zinc Deficiency by TPEN Treatment Decreased HT-22 Cell Viability and Induced Autophagy

**Figure 1:**
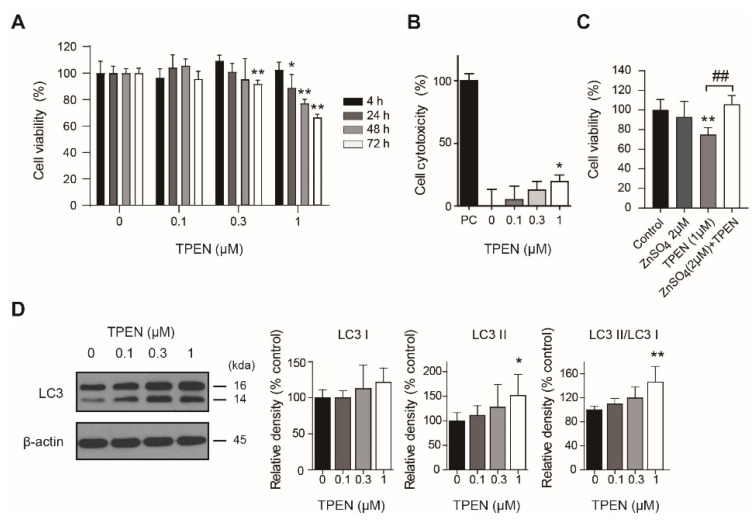
TPEN-induced cytotoxicity and autophagy in HT-22 cells. (**A**) HT-22 cell viability was measured after TPEN treatment for 4, 24, 48, and 72 h using the CCK-8 assay (*n* = 6). (**B**) The cytotoxicity of TPEN-treated cells was measured with the LDH assay (*n* = 6). PC means positive control. (**C**) The cell viability was measured using the CCK-8 assay after treatment of TPEN only or cotreatment with TPEN and zinc sulfate for 72 h (*n* = 6). (**D**) LC3-I and LC3-II levels were measured using western blotting. Values represent the mean ± SD. * *p* < 0.05, ** *p* < 0.01 versus control (0 μM group), ^##^ *p* < 0.01 between groups.

First, to confirm the effect of TPEN on cell viability, we treated HT-22 cells with TPEN at various concentrations (0, 0.1, 0.3, and 1 μM) and performed a Cell Counting Kit-8 (CCK-8) assay. The experimental results showed that TPEN at concentrations of 0.3 μM (72 h, 91.7 ± 1.2% of control group, *p* < 0.01) and 1 μM (24 h, 88.8 ± 4.1% of control group, *p* < 0.05; 48 h, 77.2 ± 1.2% of control group, *p* < 0.01; 72 h, 66.4 ± 1.0% of control group, *p* < 0.01) significantly reduced the viability of HT-22 cells (Figure 1A). To further clarify the reason for the decrease in cell viability, we measured the cytotoxicity of TPEN using an LDH assay. In the assay result, cytotoxicity significantly increased when treated with 1 μM TPEN for 72 h (*p* < 0.05, Figure 1B). Furthermore, zinc sulfate cotreatment restored the TPEN-induced decrease in cell viability (*p* < 0.01; Figure 1C).

To determine the effect of TPEN treatment on autophagic activity, we measured LC3-I and LC3-II protein levels by western blotting after TPEN treatment in HT-22 cells. After TPEN treatment, LC3-II levels (1 μM, 151.5 ± 17.5% of control group, *p* < 0.05) and the LC3-II to LC3-I ratio (1 μM, 146.6 ± 11.5% of control group, *p* < 0.01) significantly increased (Figure 1D).

### 2.2. Zinc Deficiency by TPEN Treatment Changed Autophagy-Related Signaling

To determine the mechanism by which TPEN treatment affects autophagy-related signaling, we treated TPEN for 24 h and measured AMPK and phospho-AMPK levels using western blotting. We also performed real-time PCR and western blotting analyses on TPEN-treated HT-22 cells (24 h) and measured SIRT1 and mTOR mRNA and protein levels, respectively.

**Figure 2:**
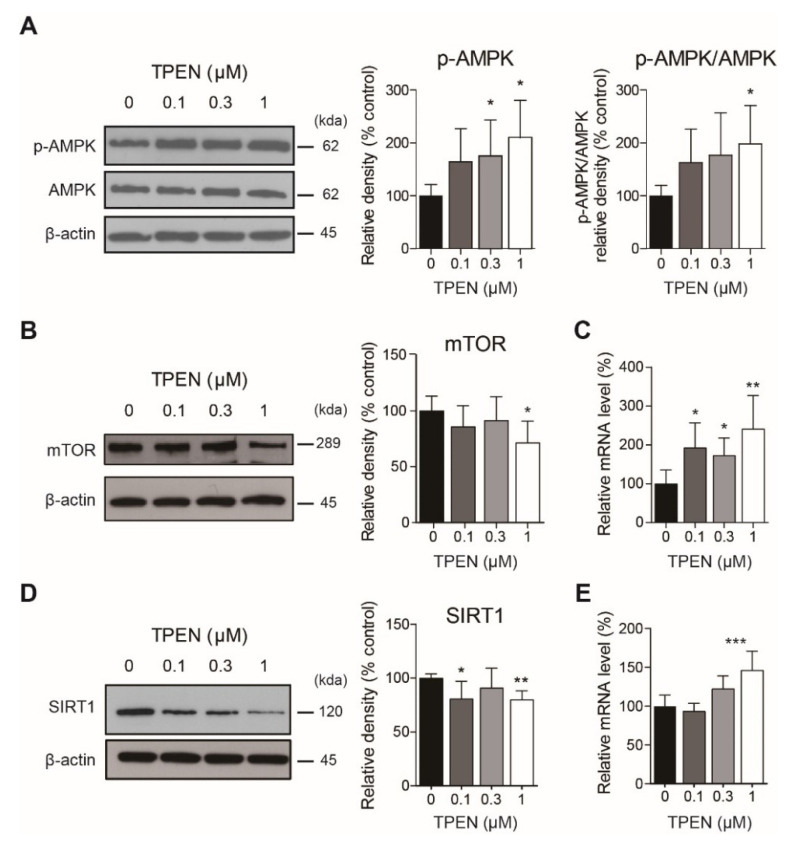
TPEN treatment changed mRNA and protein levels of autophagy-related genes. (**A**) AMPK and phospho-AMPK levels were measured using western blotting in TPEN-treated HT-22 cells. (**B**) mTOR protein level was measured using western blotting after TPEN treatment. (**C**) mTOR mRNA level was measured by real-time PCR. (**D**) SIRT1 protein level was measured using western blotting. (**E**) SIRT1 mRNA level was measured by real-time PCR. In A, B, and D, the left panel is a representative blot image, and the right panel is a bar graph. Values represent the mean ± SD. * *p* < 0.05, ** *p* < 0.01, and *** *p* < 0.001 versus control (0 μM group).

Western blot results showed that TPEN treatment significantly increased phospho-AMPK level (0.3 μM, 175.0 ± 27.9% of control group, *p* < 0.05; 1 μM, 210.1 ± 28.8% of control group, *p* < 0.05) and the ratio of phospho-AMPK to total AMPK (1 μM, 197.9 ± 29.7% of control group, *p* < 0.05) (Figure 2A). TPEN treatment significantly decreased mTOR (1 μM, 71.4 ± 8.6% of control group, *p* < 0.05) and SIRT1 (0.1 μM, 80.9 ± 6.6% of control group, *p* < 0.05; 1 μM, 80.2 ± 3.3% of control group, *p* < 0.01) protein levels (Figure 2B,D). Phospho-mTOR levels were not significantly altered by TPEN treatment (data not shown). In contrast, TPEN treatment increased the mRNA expressions of mTOR (0.1 μM, 192.9 ± 63.9% of control group, *p* < 0.05; 0.3 μM, 172.8 ± 45.2% of control group, *p* < 0.05; 1 μM, 241.5 ± 85.8% of control group, *p* < 0.01) and SRIT1 (1 μM, 146.0 ± 10.1% of control group, *p* < 0.001) (Figure 2C,E).

### 2.3. Inhibition of TPEN-Induced Autophagy by Chemical Inhibitors (3-MA, Chloroquine, Bafilomycin A1) Altered TPEN-Induced Cytotoxicity in HT-22 Cells

**Figure 3:**
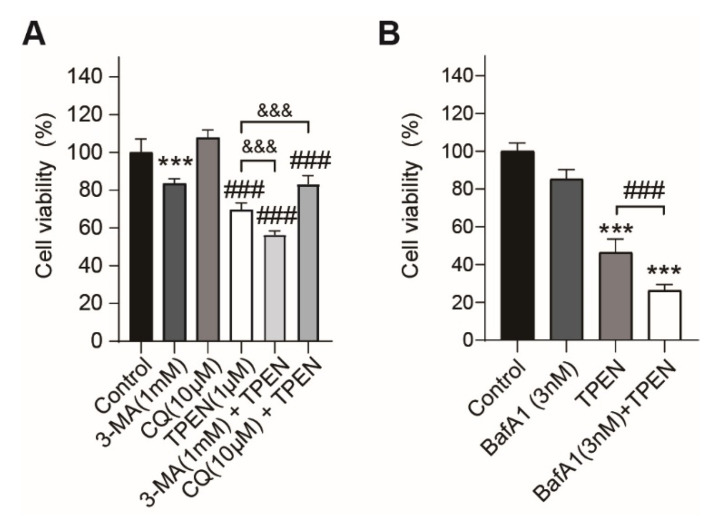
Treatment with autophagy inhibitors altered the cell viability of TPEN-treated HT-22 cells. The cell viability was measured using the CCK-8 assay after treatment of TPEN only or cotreatment with TPEN and autophagy inhibitors for 72 h (*n* = 6). (**A**) Cell viability was measured in HT-22 cells cotreated with TPEN, 3-MA, and chloroquine. (**B**) Cell viability was measured in HT-22 cells cotreated with TPEN and BafA1. Values represent the mean ± SD. In A, ***, ### *p* < 0.001, versus control groups, &&& *p* < 0.001 versus TPEN-treated group. In B, *** *p* < 0.001, versus control groups, ### *p* < 0.001 versus TPEN-treated group. CQ: chloroquine.

Treatment with 3-MA (1 mM) and chloroquine (10 μM), inhibitors that block the early and late autophagy phases, decreased cell viability in 1 mM 3-MA-treated cells (83.4 ± 1.1% of control group, *p* < 0.001, Figure 3A). When we cotreated 3-MA and chloroquine into 1 μM TPEN-treated HT-22 cells, 3-MA cotreatment worsened the TPEN-induced decrease in cell viability (56.5 ± 0.8% of the control group, *p* < 0.001), and chloroquine cotreatment restored the effect of TPEN (83.4 ± 1.8% of the control group, *p* < 0.001) (Figure 3A).

Treatment with the autophagy inhibitor Baf A1 at a concentration of 3 nM did not significantly affect the cell viability (Figure 3B). When we added 3 nM Baf A1 to 1 μM TPEN-treated cells, the decrease in cell viability by TPEN worsened in the group treated with Baf A1 and TPEN (TPEN group: 46.4 ± 7.1% of the control group, TPEN plus BafA1 group: 26.4 ± 3.1% of control group, *p* < 0.001) (Figure 3B).

## 3. Discussion

In this study, we demonstrated that zinc deficiency induces autophagy and changes AMPK and mTOR signaling in HT-22 cells, a hippocampal neuronal cell line.

Autophagy is generally considered to have a cytoprotective effect because when a cell is stressed, it immediately supplies the necessary nutrients by breaking down the intracellular components. Autophagy is an intracellular process that maintains neuronal viability and plays a pivotal role in neuronal homeostasis in neurodegenerative diseases by degrading misfolded proteins and damaged organelles [18]. However, cytotoxicity and cell death may occur when the autophagy activity exceeds a certain threshold [19]. The precise mechanisms underlying excessive autophagy remain unclear. The mechanism by which autophagy protects hippocampal neurons from zinc deficiency has not yet been elucidated. A study in yeast showed that zinc deficiency caused autophagy, and genetic blockade of autophagy inhibited cellular growth [11,16]. Metallothioneins (MTs), proteins that bind zinc and buffer intracellular zinc levels, are degraded in lysosomes [20,21]. Other studies have confirmed that lysosomal zinc is transported into the cytosol by the zinc transporters Zrt- and Irt-like protein 8 (ZIP8) and transient receptor potential cation channel mucolipin subfamily member 1 (TRPML1) [22,23]. Zinc recycling occurs through MTs degradation and zinc transport by autophagy, and intracellular zinc levels can be restored. Notably, intracellular free zinc levels reportedly decrease when drugs or genetic methods are used to inhibit autophagy [15,24].

After treatment with 3-MA, or bafilomycin A1, and autophagy inhibitors, together with TPEN, our experimental results suggested that autophagy has a cytoprotective effect in a zinc deficiency state. Although chloroquine is known to inhibit lysosomal function and act as an autophagy inhibitor, it reportedly enhances zinc uptake [25]. In this study, we also observed that treatment with 10 M chloroquine induced cell proliferation and attenuated the decrease in cell viability following TPEN treatment.

AMPK signaling is activated by sensing the level of available energy in the cell. Activated AMPK increases catabolism and decreases anabolism. In this study, zinc deficiency induced by TPEN treatment activated AMPK via AMPK Thr 172 phosphorylation. The AMPK activation mechanisms include changes in the ATP-to-AMP ratio and upstream kinases, such as tumor suppressor liver kinase B1 (LKB1) and calcium/calmodulin-dependent protein kinase kinase 2 (CAMKK2) [8]. Mechanisms related to AMPK activation causing autophagy have been studied, including unc-51-like kinase 1 (ULK1) activation by its phosphorylation, beclin1 phosphorylation, and p53 attenuation [9,26,27,28]. Metformin, an antihyperglycemic agent which activates neuronal AMPK at Thr172, reportedly restored the decrease of hippocampal synaptic density in the sevoflurane-induced memory impairment animal model by activating autophagy [29]. Furthermore, TPEN treatment decreased protein levels of mTOR and SIRT1. As the mRNA expression of both genes increased, the decrease in protein amount may be due to an increase in degradation. There are few studies on changes in the amount of mTOR protein associated with autophagy. One study showed that the mechanism of autophagy activation by a halofuginone-induced amino acid response in cancer cell lines is the proteasomal degradation of mTOR [30]. A decrease in mTOR protein levels due to zinc deficiency may also occur through this mechanism. SIRT1 is a well-known autophagy regulator. Caloric restriction increases SIRT1 expression [31], which in turn positively regulates autophagy [32,33,34]. One study showed that reactive oxygen species reduce the amount of SIRT1 protein [35], and another study showed that in the aging process, SIRT1 is a substrate of the autophagic process [36]. The reduction of SIRT1 protein levels by TPEN may be an acute negative feedback mechanism to prevent excessive autophagy. Follow-up studies will elucidate the significance of the zinc deficiency-induced decrease in SIRT1 and mTOR protein levels on neuronal function.

There are limitations to this study. TPEN does not selectively chelate zinc but affects cellular iron and copper levels. However, the experimental results showed that zinc sulfate treatment completely restored the TPEN-induced decrease of cell viability, and treatment with chloroquine, a zinc ionophore, also significantly restored cell viability [37]. Therefore, we can suggest that zinc deficiency may account for a large part of the effect of TPEN on cell viability. In addition, when we used autophagy inhibitors to investigate the role of TPEN-induced autophagy, autophagy inhibitors reduced cell viability at a particular concentration in the HT-22 cell line (data not shown). Therefore, we treated inhibitors at concentrations that did not significantly affect cell viability (bafA1) or had a statistically significant but modest effect (3-MA).

In conclusion, this study revealed that zinc deficiency induces autophagy and alters autophagy-related signaling in hippocampal neurons. In addition, we demonstrated that zinc deficiency-induced autophagy had a cytoprotective function in neurons.

## 4. Materials and Methods

### 4.1. Cell Culture

HT-22 mouse hippocampal neuronal cells were purchased from Millipore (Burlington, MA, USA). Cells were maintained in 150-mm Petri dishes with Dulbecco’s modified Eagle medium (DMEM, Lonza, Basel, Switzerland) supplemented with 10% fetal bovine serum (FBS, Alphabioregen, Boston, MA, USA) and 1% penicillin-streptomycin (Gibco, Waltham, MA, USA) in an atmosphere of 5% CO_2_ at 37 °C. We performed a subculture when the cells reached 80–90% confluency.

### 4.2. Drug Treatments

TPEN (#P4413), zinc sulfate (#83265), compound C (#HY-13418A), 3-methyladenine (#M9281, 3-MA), bafilomycinA1 (#B1793, BafA1), and chloroquine (#C6628) (Sigma Aldrich, St. Louis, MO, USA) were purchased. TPEN was dissolved in ethanol to obtain a stock solution and diluted to the final working concentration with Dulbecco’s phosphate-buffered saline (DPBS). We dissolved zinc sulfate, 3-MA, and chloroquine in nuclease-free water (Invitrogen, Waltham, MA, USA) and BafA1 in dimethyl sulfoxide (DMSO, Sigma Aldrich, St. Louis, MO, USA). We treated various concentrations of drugs or vehicle solution (ethanol in DPBS or DMSO) into HT-22 cells 24 h after seeding.

### 4.3. Cell Viability Assay

HT-22 cells were seeded in 96-well plates (1 × 10^5^ cells/100 μL). Twenty-four hours after seeding, the cells were treated with various concentrations of TPEN, zinc sulfate, 3-MA, and chloroquine for 24, 48, and 72 h. The viability of HT-22 cells was measured using the Cell Counting Kit-8 reagent (#CK04-011, CCK-8, Dojindo Molecular Technologies, MD, USA) according to the manufacturer’s instructions. After adding 10 μL of CCK-8 solution, the cells were incubated at 37 °C for 1 h. The absorbance was then measured using a MultiSkanTM microplate photometer (Thermo Fisher Scientific, Waltham, MA, USA) at 450 nm.

### 4.4. Lactate Dehydrogenase (LDH) Assay

The cytotoxicity of TPEN in HT-22 cells was measured using an LDH detection kit (#MK401, Takara, Kusatsu, Japan). Cells were seeded on a 96-well plate (1 × 10^5^ cells/100 μL) and treated with control, 0.1, 0.3, and 1 μM TPEN after 24 h. After 72 h of TPEN treatment, new wells were treated with 1% Triton X-100 (LPS solution, Daejeon, Korea) for 10 min as a positive control, and the medium was centrifuged for 3 min to extract the supernatant. The supernatant was transferred to a new 96-well plate, and the solution contained in the LDH kit was added. The supernatant was incubated with reaction mixtures in the dark for 30 min, and the absorbance was measured with a MultiSkanTM microplate photometer (Thermo Fisher Scientific, Waltham, MA, USA) at 490 nm. We analyzed the LDH assay results using the values of the control wells as negative controls.

### 4.5. Quantitative Real-Time PCR (qRT-PCR) Analysis

We isolated mRNA from HT-22 cells using an RNeasy Mini Kit (#74104, Qiagen, Hilden, Germany). We then measured the concentration of mRNA using Nanodrop Lite (Thermo Fisher Scientific, Waltham, MA, USA) and synthesized cDNA (500 ng/μL) using an iScript first-strand cDNA synthesis kit (Bio-Rad, Hercules, CA, USA). We performed qRT-PCR using iQTM SYBR^®^ Green Supermix (Bio-Rad, Hercules, CA, USA) with a CFX96 Touch Real-Time PCR Detection System (Bio-Rad). The relative quantification of mRNA expression was normalized to glyceraldehyde 3-phosphate dehydrogenase (GAPDH) (internal control). The primers used were GAPDH-F:5′-GAG TCA ACG GAT TTG GTC GT-3′, GAPDH-R:5′-GAT CTC GCT CCT GGA AGA TG-3′; SIRT1-F:5′-TGT TGG TTG ACT TCA TCT TCC TT-3′, SIRT1-R:5′-TCC AAT GGC TTT TGA AAA CTT TA-3′; mTOR-F:5′-ACT TGA CAT CAT CCG AGC AG-3′; mTOR-R:5′-CCA ACA TGC TGA TGC ACG-3′. Target genes were amplified under the following conditions: 40 cycles of initial denaturation at 95 °C for 3 min, followed by annealing at a suitable temperature (55–60 °C) for each product for 1 min.

### 4.6. Western Blot Analysis

After drug treatment, whole-cell lysates were prepared from HT-22 cells in RIPA buffer (150 mM sodium chloride, 1% Triton X-100, 0.1% SDS, 1% sodium deoxycholate, 50 mM Tris-HCl pH 7.5, 2 mM EDTA, sterile solution) with proteinase and phosphatase inhibitors (ATTO, Tokyo, Japan). The protein samples (20 μg/well) were loaded and separated on 8–15% polyacrylamide gels for 2 h. The proteins (2 h, 100 V) were then transferred onto a polyvinylidene fluoride (PVDF) membrane (Millipore, Burlington, MA, USA). The membranes were blocked with 5% skim milk in Tris-buffered saline pH 7.4 (TBS-T buffer) for 1 h to overnight at 4 °C and then incubated with specific primary antibodies at 4 °C overnight. The following primary antibodies were used: anti-β-actin, anti-mTOR, anti-p-mTOR, anti-SIRT1, anti-LC3, anti-p-AMPK, and anti-AMPK (Cell Signaling Technology, Beverly, MA, USA). We included detailed information on primary antibodies in Supplementary Materials Table S1. After incubation, the membrane was washed thrice with TBS-T and conjugated to mouse or rabbit secondary antibodies (#31430, #31460, Invitrogen, Carlsbad, CA, USA) at room temperature for 1 h. Protein bands were visualized using Forte solution (Millipore, Burlington, MA, USA) or Femto solution (Thermo Fisher Scientific, Waltham, MA, USA) after washing the membranes thrice with TBS-T buffer. The relative densities were quantitatively analyzed using ImageJ 1.53e software (NIH, Bethesda, MD, USA).

### 4.7. Statistical Analysis

All data are presented as mean ± standard deviation (SD). Statistical analyses were performed using SPSS version 20 (IBM, Chicago, IL, USA) for Windows. The statistical significance of differences between groups was evaluated by Student’s *t*-test and one-way analysis of variance (ANOVA) followed by Tukey’s multiple range test. *p* < 0.05 was considered statistically significant.

## Data Availability

The authors declare that all required data have been presented in the manuscript. The datasets did not contain any software code needing to be archived.

## Supplementary Materials

The supporting information can be downloaded at: https://www.mdpi.com/article/10.3390/ijms23158811/s1.
