# Supplementary material for: Zinc Deficiency Induces Autophagy in HT-22 Mouse Hippocampal Neuronal Cell Line
Source: Int J Mol Sci. 2022 Aug 8;23(15):8811. doi: 10.3390/ijms23158811 (PMC9369147; doi:10.3390/ijms23158811)
Supplement: Supplementary file 1 [file ijms-23-08811-s001.zip › ijms-1824763-supplementary.pdf]

Supplementary Table S1. List of antibodies used in Western blot

Antibody	Species	Dilution	Company (Catalog#)
β-actin	Rabbit	1:3000	Cell signaling (4970)
SIRT1	Mouse	1:1000	Cell signaling (8469)
LC3	Rabbit	1:1000	Cell signaling (4108)
p-AMPK	Rabbit	1:1000	Cell signaling (2531)
AMPK	Rabbit	1:1000	Cell signaling (2603)
mTOR	Rabbit	1:1000	Cell signaling (2983)
p-mTOR	Rabbit	1:1000	Cell signaling (2971)
